# Brain Metastatic Lung Cancer Patients: A Multitarget Therapeutic-Supportive Strategy with Anti-STAT3 Silibinin

**DOI:** 10.3390/neurosci6040131

**Published:** 2025-12-18

**Authors:** Elisa Roca, Elena Roca, Alessandra Cucinella, Giorgio Madonia, Giovanni Centonze, Fiorella Lombardo, Licia Martinelli, Maria Elisa Damiani, Antonio Santo

**Affiliations:** 1 Thoracic Oncology—Lung Unit, Pederzoli P. Hospital, 37019 Peschiera del Garda, Italy; cucinella.alessandra@gmail.com (A.C.); giorgiomadonia92@gmail.com (G.M.); fiorella.med@gmail.com (F.L.); licia.martinelli@ospedalepederzoli.it (L.M.); mariaelisa.damiani@ospedalepederzoli.it (M.E.D.); asanto953@gmail.com (A.S.); 2 Neurosurgery Department, Istituto Ospedaliero Fondazione Poliambulanza, 25124 Brescia, Italy; elena.roca@poliambulanza.it; 3 Food Safety and Nutrition Service—S.I.A.N., Department of Prevention, Azienda Sanitaria Locale ASL CN2, 12051 Alba-Bra, Italy; giovanni.centonze6@gmail.com

**Keywords:** lung cancer, brain metastases, STAT3 (signal transducer and activator of transcription 3), silibinin, quality of life

## Abstract

Background: Innovative treatments for lung cancer patients have significantly improved their lives. Therefore, patients who develop brain metastases are more likely to require management of quality of life (QoL) by reducing pathological decline in brain function. New therapeutic strategies have allowed us to manage brain metastases, thanks to the ability to cross the blood–brain barrier. Moreover, new molecules have been designed as adjuvants to standard treatments for the management of cancer patients with brain metastases. Methods: We implemented a descriptive, observational, retrospective study. Therefore, we consecutively collected the data of eighty-six (*N*  =  86) patients admitted to our department (April 2020–April 2025) diagnosed with brain involvement in a thoracic neoplasm and treated with silibinin, in association with standard treatment. The main endpoint of our analysis is to define the safety profile of silibinin and to evaluate its eventual benefits in terms of QoL. Results: Silibinin was well tolerated (only one mild adverse event was reported); furthermore, patients taking silibinin had a good quality of life that was maintained over a long period of time, and in some cases, an improvement in neurological symptoms and overall patient well-being was also documented. Conclusions: Our study is the first collection of a large number of lung cancer patients with brain metastasis taking silibinin, which is very well tolerated and allows patients to maintain a good QoL.

## 1. Introduction

Global life expectancy is still strongly affected by cancer mortality, which is steadily increasing [1]. Among the most common cancers, lung cancer remains the leading cause of cancer death worldwide, despite new therapeutic frontiers. Tobacco is the main etiological factor in lung carcinogenesis, but other risk factors (such as occupational exposures, air pollution, genetic susceptibility and poor diet) may have a role along with tobacco smoking [2,3,4].

Lung cancer has a five-year survival rate of 15% and about 70% of patients present with locally advanced or metastatic disease at the time of diagnosis [3]. Patients with stages I to II NSCLC can benefit from surgical resection, whereas chemotherapy is beneficial for patients with metastatic disease, and concurrent chemotherapy and radiation is indicated for stage III lung cancer. The introduction of new anti-cancer agents such as angiogenesis, epidermal growth factor receptor inhibitors, personalized biological therapies and immunotherapy has drastically improved the lives of these patients, with a significant increase in the survival rate [3,5].

With the improvement in life expectancy, the number of patients developing brain metastases is increasing. Thoracic oncologists are managing large numbers of patients with this condition, thus influencing prognosis and quality of life. There are some studies that analyze the role of metabolic plasticity and immunometabolism in the tumor microenvironment during metastasis: this suggests the hypothesis of the presence of an immunosuppressive niche in brain metastases. Thus, tumor glycolysis is probably associated with brain metastasis and immune evasion [6,7].

Moreover, thanks to new systemic therapeutic approaches, such as targeted biological therapy and antiangiogenic drugs, allowing us to manage brain metastases without resorting to standard treatments (radiotherapy and/or neurosurgery) and gives us the ability to cross the blood–brain barrier and reach the tumoral cells even in this site [8,9,10,11,12,13].

Given the higher survival rates among patients with secondary brain tumors, it is essential to stabilize their quality of life by reducing the pathological decline in brain function associated with the disease progression as much as possible. In this landscape, new molecules have been developed and could be considered to be adjuvants to standard treatments for the management of cancer patients with brain metastases, designing more effective and synergic therapeutic approaches.

Out of them, STAT3 (signal transducer and activator of transcription 3) inhibitors, flavonoid compounds extracted from the seeds of Silybum marianum, could play a role in reducing the toxicity of ongoing anti-cancer treatments and increasing the therapeutic potential of innovative antitumor drugs. Silibinin is a STAT3 inhibitor which seems to have an adjuvant role in antitumor pathways, although its own molecular mechanism against cancer cells remains not fully explained: it could probably decrease the tumor volume and delay the tumor tissue development. Some authors performed in vitro Western blot analysis and their results indicate that the possible molecular mechanism involved inhibiting cleaved CASP3 (caspase 3), MMP3 (Matrix Metallopeptidase 3), SRC (Proto-oncogene tyrosine-protein kinase Src), MAPK10 (mitogen-activated protein kinase 10) and CDK6 (Cyclin-dependent kinase 6), and activating PPARα (Peroxisome proliferator-activated receptor alpha) and JAK (Janus Kinase) [2].

Silibinin is the major constituent of milk thistle extract: it is found in its fruits, seeds and leaves [14,15,16,17,18]. Silybum marianum, also known as milk thistle, grows in America, Australia, Southern Europe, North Africa and parts of Asia. Milk thistle belongs to the Asteraceae family and, since ancient times, has been proven to reduce liver toxicity and blood lipid, as well as increasing milk production in lactating mothers, in addition to its antitumor effects [19,20,21,22]. For this reason, several studies have investigated the mechanisms of silibinin: a promising molecule with demonstrated anti-cancer effects, based on the activation of specific molecular pathways [23,24,25,26,27].

Indeed, STAT3 is a key protein that has the role of a transcriptional factor, influencing cell proliferation and solid tumor metastasization through tumor microenvironment involvement. Moreover, STAT3 dysregulation seems to be one of the basic steps of oncogenesis. Further data suggest that STAT3 inhibition would also contribute to reducing immunosuppression and increasing the immune infiltrate in the tumor, as well as reducing cancer-related inflammation with consequent clinical improvement in the patient: including a reduction in pain and fatigue and improvement in neurological function and quality of life.

This evidence led us to investigate the role of these molecules on cancer patients, with a dedicated review of the literature, which we offer here.

Furthermore, we have also evaluated the impact of STAT3 inhibitors in real life, integrating silibinin into our clinical practice; therefore, we summarize our experience with this molecule in lung cancer patients with central nervous system (CNS) involvement. We performed a descriptive, observational, retrospective study. The main endpoints of our analysis are to define the safety profile of silibinin and to evaluate the eventual benefits derived from this molecule in terms of QoL.

### 1.1. Focus on Silibinin: From Theory

#### 1.1.1. Role of Silibinin in Fighting Cancer

Silibinin is a STAT3 inhibitor; more specifically, it selectively inhibits the signal transducer and activator of transcription 3.

STAT3 is an intracellular transcription factor activated by JAK [6,23]. Dysregulation of the JAK/STAT3 signaling pathway appears to lead to alterations in the cell cycle. Indeed, STAT3 appears to be involved in inflammation, uncontrolled cell proliferation, apoptosis, metastasis and immunological tolerance.

This is the reason why STAT3 inhibitors are considered to be innovative weapons against a potential therapeutic target for various types of tumors, since this pathway is strongly involved in carcinogenesis [28].

Out of the STAT3 inhibitors, silibinin has been shown to have specific anti-inflammatory, antioxidant, antiproliferative, antimetastatic and anti-angiogenic action, and thus, it could have interesting antitumor activity against several types of cancers. In more detail, STAT3 is involved in several signaling pathways underpinning cancer development and progression, and in many pathogenic pathways related to inflammation and cancerization [29,30,31,32].

Indeed, through PI3K/Akt (phosphatidylinositol 3-kinase/serine/threonine kinase), NF-κB (Nuclear Factor kappa-light-chain-enhancer of activated B cells), Wnt/β-catenin (Wingless-related integration beta—catenin), and MAPK pathways, it seems to be involved in cancer cell proliferation and in promoting apoptosis. In addition to programmed cell death, silibinin also plays a cytotoxic role, acting on the CCK-8 (Cell Counting—8) pathway and on the Zein-β-cyclodextrin complex [26,27].

By acting on reactive oxygen species (ROS) generation–induction, silibinin also appears to be linked to the reduction in cell proliferation, through a calpain-dependent pathway, to the epithelial–mesenchymal transition and to cell migration [33,34,35,36,37].

Some studies have demonstrated an effective function on Epidermal Growth Factor Receptor (EGFR) as well, suggesting a possible future role for silibinin in combination with anti-EGFR-targeted therapies [28].

Silibinin appears to be effective on the central nervous system too. Indeed, many studies have proven the efficacy of this molecule on brain tumors [6,38,39,40].

In addition to acting on primary tumors of the central nervous system, silibinin may play an important role in brain metastases arising from other tumors [41,42,43,44]. In particular, several studies have shown that STAT3 is involved in the development of brain metastases through interaction between endothelial cells and tumor cells; indeed, brain metastases activate the STAT3 pathway in reactive astrocytes and influence the cellular microenvironment, exerting a modulatory effect on the immune system. In this context, STAT3 inhibition could be an effective therapeutic frontier, suppressing the brain metastases of solid tumors [44,45,46,47,48,49].

#### 1.1.2. Silibinin: Safety Profile

Since silibinin is a natural compound, derived from milk thistle, it seems to have a favorable safety profile, as well as synergistic effects with conventional therapies. Studies that have analyzed the effects of silibinin in humans have documented very mild and uncommon gastrointestinal alterations, such as loss of appetite, nausea, vomiting, abdominal swelling, abdominal pain, flatulence and diarrhea. Other symptoms reported by patients, such as asthenia, headache and dizziness, may be non-specific, especially considering simultaneous treatments [40].

In any case, the side effects described as secondary to the use of silibinin are very rare and mild, and the majority of researchers who have analyzed silibinin have not documented any of them. For example, Zhu et al. tested the oral use of Silymarin (140 mg three times a day for 28 days) without any harmful effects; Di Pierro et al. documented no side effects in a group of 50 healthy breastfeeding women who received Silymarin daily (oral dose of 420 mg for 63 days) [50,51]. In addition, Valentova et al. confirmed no toxicity after administration of silibinin in 22 healthy volunteers (oral dose of 400 mg per day) [52].

The absence of significant side effects or toxicity associated with this molecule appear to be regardless of the administration route [51,52,53].

The safety of this adjuvant supplement for standard therapies has also been tested in different types of neoplasms: prostate cancer, head and neck cancer and colorectal cancer [54,55,56,57].

Vidlar et al. demonstrated that Silymarin, in association with selenium, is effective in preventing the progression of prostate cancer and does not cause toxicity at a dosage of 570 mg per day [58].

Given the good tolerability profile of silibinin in various types of tumors and in combination with standard therapies, and since at therapeutic dosage, this molecule may rarely appear to cause mild gastrointestinal side effects [29,59], we decided to test silibinin in patients affected by thoracic cancer and brain metastases.

## 2. Materials and Methods

From April 2020 to April 2025, we consecutively analyzed the data of patients who were admitted to our Department of Thoracic Oncology—Lung Unit and diagnosed with metastatic brain involvement from lung cancer who assumed supplements in association with standard oncological treatment based on the histological diagnosis. Among these patients, we collected the data of those who were taking silibinin at a dose of 800 mg (one tablet) twice a day for the first month, then 1 tablet daily continuously, with active treatment. Informed consent for participation and publication of this paper was obtained from all subjects involved in the study. The study was conducted in accordance with the Declaration of Helsinki; no review board/ethic committee approval was necessary, since the patients took silibinin as part of their home treatment. Our study is a descriptive, observational, retrospective study; the main endpoints of our analysis are to define the safety profile of silibinin and to evaluate the potential benefits derived from this molecule in terms of QoL.

To analyze the toxicity profile of silibinin, we collected all available information relating to side effects described by patients during each medical examination carried out as a standard routine for anti-cancer treatments. In particular, as per clinical practice, we carried out all the relevant tests to assess the tolerance profile of silibinin: vital sign measurements, physical examination, laboratory analysis, questionnaires relating to patient-reported outcomes and patient diaries. We have collected all this information and recorded it according to the National Cancer Institute Common Terminology Criteria for Adverse Events (CTCAE—V.5.0), which was used for grading toxicities: adverse events (AEs) and serious AEs (SAEs) [33].

In order to determine whether there was a change in quality of life during the lives of the patients studied, we applied validated questionnaires that were routinely used in clinical practice. In particular, we used the following: the EORTC QLG Core questionnaire (EORTC QLQ-C30), the EQ-5D 5-level test (EQ-5D-5L) and the visual analog scale [34,35,36].

The EORTC QLQ-C30 was developed in 1993 as a result of a series of multidisciplinary studies that identified the need to collect data on quality of life. It is a test consisting of 30 items about different aspects of the cancer patients’ QoL.

The EQ-5D-5L is another questionnaire that we administered to our patients. It consists of a short test created in 2009 to improve sensitivity and reduce ceiling effects compared to the EQ-5D-3L. The EQ-5D-5L can be divided into two parts: the first included specific signs and symptoms (mobility, self-care, usual activities, pain/discomfort and anxiety/depression), described according to 5 levels (no problems, mild problems, moderate problems, severe problems and extreme problems), and the second one consisted of a quality of life visual analog scale (QoL VAS) for the assessment of patients’ health, using a quantitative measure of health outcome to obtain excellent reliability and a realistic understanding of the patient’s own judgement.

Moreover, we applied a test based on neurological symptoms—the (EORTC) QLQ-BN20—to test in detail the quality of life of our patients who had brain metastases from lung cancer. This questionnaire, designed in 1961 and further validated in 2010, assesses the health-related quality of life (HRQoL) of patients with brain tumors [37,60].

Only patients who received EORTC questionnaires at least two times, at baseline and at the time of the first reevaluation, have been considered to be eligible and were included in the final analysis, in order to evaluate the clinical evolution of neurological symptoms. Patients not included were lost at follow-up or had early adverse events related to oncological disease. This exclusion has reduced the sample size and must be cited as a limit of our study.

### Statistical Analysis

This is an exploratory, descriptive and observational study. Data were analyzed using descriptive statistics. Comparisons of the QLQ-BN20, QLQ-C30 and EQ5D questionnaires and the QoL visual analog scale, among different time administrations (baseline condition and the latest available assessment), were evaluated using the nonparametric Wilcoxon test for continuous variables. Given the descriptive and exploratory nature of the study, the Wilcoxon tests were performed to explore potential patterns or trends over time. Accordingly, no adjustment for multiple testing was applied. Overall survival (OS) was assessed from the date of diagnosis to the date of death or last follow-up. The statistical analyses were performed using the R environment for statistical computing and graphics (R Foundation, Vienna, Austria, Version 4.3.3).

## 3. Results: Silibinin—To Practice

### 3.1. Patients’ Clinical and Biological Features

Eighty-six (*N*  =  86) lung cancer patients with brain metastasis who were admitted to our Thoracic Oncology—Lung Unit between April 2020 and April 2025 and received silibinin as a supplement were consecutively studied.

Out of them, 41 were male (47.7%) and 45 female (52.3%); the median age was 69.5 years.

The ECOG performance status (PS) was 0 in 17 (19.8%) patients, 1 in 41 (47.7%) of cases and 2 in 28 (32.6%).

All patients in our lung unit adhere to a nutritional care program; therefore, we were also able to collect data relating to the BMI (body mass index): the average BMI was 24.08 with a median of 23.67.

With regard to smoking habits, 19 (22.1%) patients were current smokers, 40 (46.5%) were ex-smokers and 27 (31.4%) patients had never smoked. The average tobacco exposure of the analyzed population was 29.43 packs/years (median 30).

**Figure 1:**
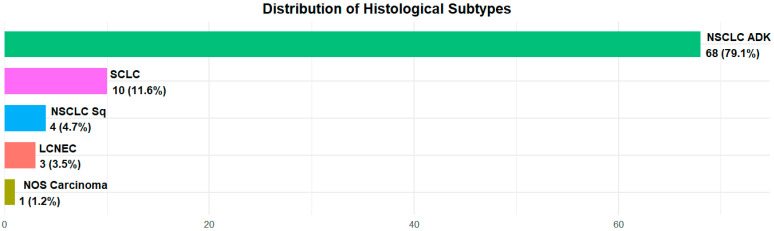
Histology distribution in the overall population.

All patients were suffering from lung neoplasia and among them, 73 (84.9%) had a diagnosis of non-small cell lung cancer (NSCLC)—68 (79.1%) were adenocarcinomas and 4 patients (4.7%) were squamous carcinomas; 10 (11.6%) were affected by small cell lung cancer (SCLC); 3 (3.4%) by neuroendocrine neoplasia: 2 (2.3%) by neuroendocrine lung cancer; 1 (1.2%) by large cell lung cancer and only 1 (1.2%) patient was diagnosed with Nos (non-otherwise-specified) carcinoma (Figure 1).

For NSCLC adenocarcinoma patients, biomolecular profile analysis was carried out and 26 (30.2% of total patients, 38.2% among NSCLC histology) were “mutated” (i.e., carriers of a driver mutation): 21 (30.9% of 68) had a genetic alteration of EGFR and among them, 5 carried the KRAS g12C pathogenic variant, while no patients had alterations in ALK and ROS1.

Regarding the metastatic sites, NSCLC patients had lung metastases in 21 (28.8%) cases, pleural involvement in 10 (13.7%), hepatic in 10 (13.7%), bone in 17 (23.3%), lymph node in 13 (17.8%), adrenal in 9 (12.3%), renal in 2 (2.7%), splenic in 1 (1.4%) and skin in 1 (1.4%).

All patients with neuroendocrine neoplasia (including SCLC) had extended disease (ED). Out of neuroendocrine neoplasia, all SCLC patients had lung metastatic disease: pleural in five (50%), liver in five (50%), kidney in one (10%), adrenal in four (40%), bone in two (20%) and lymph node involvement in one (10%); while neuroendocrine patients had bone metastases in one (50%) and adrenal in one (50%), all had pulmonary metastases.

**Figure 2:**
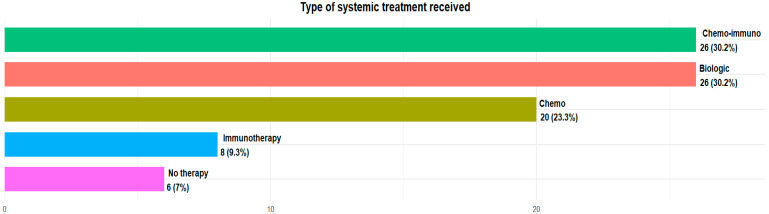
Type of systemic treatment received.

The majority of patients in our analysis were taking silibinin as a supplement to standard oncological therapy: 20 (23.3%) patients were treated with standard chemotherapy agents, 8 (9.3%) with immunotherapy alone, 26 (30.2%) with chemoimmunotherapy combination regimens and 26 (30.2%) with oral target therapy, because of their biomolecular profile. Only six (7%) were not under treatment at the moment of silibinin assumption (Figure 2).

All patients in our court were characterized by metastatic brain disease: 37 (43.0%) had only one brain lesion, 24 (27.9%) had one to four brain metastases and 22 (25.6%) had more than five lesions.

Out of them, 44 (51.2%) patients had been treated with a radiotherapy approach at the time of diagnosis: among these, 7 had undergone a second radiotherapy treatment and approximately 2 were treated further with radiotherapy.

In Table 1, we summarize the overall population characteristics.

### 3.2. Silibinin Tolerance

The main objective of our research was to analyze the tolerance of silibinin in patients with lung cancer and brain metastases, in combination with standard therapies. As per clinical practice, all our patients underwent medical examinations organized for the anti-cancer therapies they had planned or for clinical monitoring if they were only in follow-up.

Indeed, we collected all information relating to side effects as a standard routine and, in particular, we analyzed the toxicity profile of silibinin.

No alteration of vital sign measurements or laboratory analysis were documented. The physical examination was consistent with the patient’s clinical condition at the time of the visit and was never affected by the simultaneous administration of silibinin. Questionnaires relating to patient-reported outcomes and patient diaries have not detected any significant side effects related to silibinin.

We also specifically questioned patients about the main toxicities associated with STAT3 inhibitors and those documented in the literature: loss of appetite, nausea, vomiting, abdominal pain, dyspepsia, flatulence, swelling, diarrhea, headache, dizziness and fatigue. No patients showed toxicity related to silibinin intake, and only one patient reported very mild diarrhea, lasting a few days at the start of supplementation.

The toxicity profile of silibinin is therefore optimal for patients with lung cancer and brain metastases, even in combination with other anti-cancer treatments and regardless of the therapeutic strategies carried out (chemotherapy, biological therapy, immunotherapy).

### 3.3. Silibinin and Quality of Life

Another objective of our study was to assess whether silibinin intake allowed lung cancer patients with brain metastases to maintain a good quality of life throughout the course of their lives, despite the physiological decline in nervous system function.

For this reason, we analyzed our patients’ QoL in detail, administering specific questionnaires at different times: at baseline, at their first restaging follow-up evaluation and at the last reassessment (before death or at the last follow-up). The questionnaires we applied were QLQ-BN20, QLQ-C30, EQ5D and the QoL visual analog scale, and at our lung unit, questionnaires were administered as routine clinical practice.

Below is a comparison between the baseline condition and the latest available assessment.

QLQ-BN20 is dedicated to describing symptoms specifically related to alterations in the central nervous system. More specifically, it is a scale of symptoms ranging from 0 to 100, indicating a progressive worsening of the patient’s overall neurological condition. Patients treated with silibinin did not experience a statistically significant change in QLQ-BN20 scores; in addition, the median decrease from 19.16 to 16.67 indicates an absence of significant worsening, suggesting a potential benefit for maintaining patients’ symptoms (Table 2).

QLQ-C30 is a questionnaire divided into three sections: one dedicated to functions, one dedicated to symptoms and one dedicated to QoL.

The part of the QLQC30 relating to function consists of a scale ranging from 0 to 100, indicating the improvement in the patient’s general condition. The QLQC30 relating to symptoms (part II) consists of a scale in hundredths, where 0 indicates no symptoms and 100 indicates maximum deterioration. The QoL part of the QLQC30 is the third part of this questionnaire and indicates an improvement in quality of life from 0 (worst QoL) to 100 (best possible QoL).

**Figure 3:**
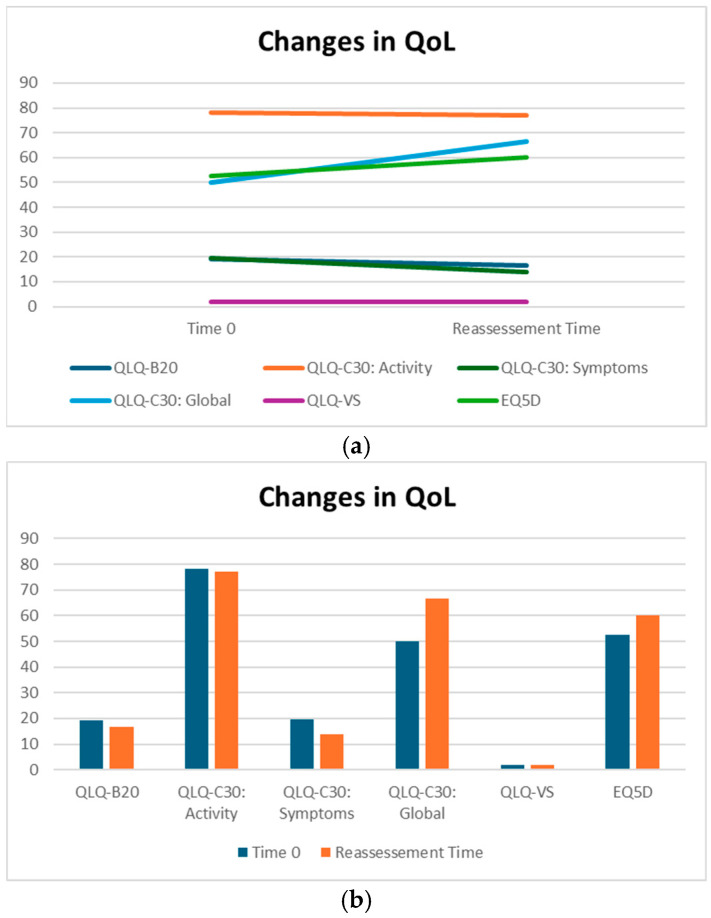
(**a**,**b**) QoL on Sillbrain: changes in questionnaire scores between baseline and QoL reassessment.

Although not statistically significant, the patients in our cohort showed a median improvement in symptom-related QLQ (part II: from 19.44 to 13.86) and overall QoL (part III: from 50 to 66.67), with no deterioration in QLQ-C30 activity (part I: from 78.12 to 77.08) despite disease progression (Table 2). Interestingly, in the subgroup of ADK patients, QoL scores tended to improve (from 58.3 to 66.7), with a nearly significant trend (*p* = 0.07, Figure 3). Considering that the functional scales of the QLQ-C30 range from 0 to 100, this finding may reflect a clinically meaningful improvement in patients’ general condition. From a clinical perspective, these results suggest a potential absence of deterioration in the patients’ overall status, even in the context of disease progression. The median QoL VAS score increased from 52.50 to 60.0; although this change was not statistically significant, the scores did not decrease significantly, suggesting that silibinin support, from a clinical point of view, may help maintain patients’ health and well-being (Table 2).

We also analyzed the data obtained from an additional questionnaire—the EQD5—which uses a scale to describe symptoms ranging from 0 (no symptoms) to 10 (worst symptoms).

The EQ5D questionnaires we collected did not show significant changes in quality of life (*p* value = 0.198) between the baseline and restaging (median 1.99 and median 2 vs. 3.25 and median 3).

Thanks to the data provided by the EQ5D questionnaire analysis, it has been demonstrated that quality of life remained good throughout the duration of treatment, with no evident deterioration in the patient’s general condition, despite the progression of the disease (from two to two) (Table 2).

**Figure 4:**
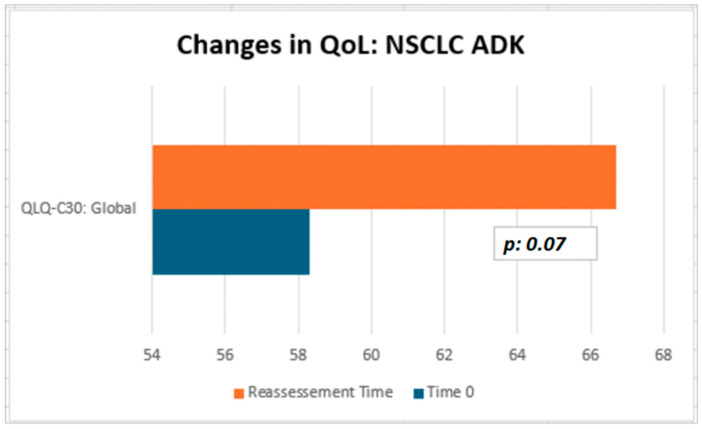
QoL on Sillbrain: changes in QLQ-C30 global questionnaire scores between baseline and QoL reassessment.

In conclusion, none of the statistical comparisons yielded *p*-values below the conventional significance threshold (*p* < 0.05). Therefore, all results should be interpreted descriptively as indicators of potential clinical trends and confirmation of the overall absence of measurable changes across timepoints. In Figure 3 and Figure 4, we summarized the overall results of QoL questionnaires.

At the time of data analysis, 29 (33.7%) patients were alive, while 57 (66.3%) had died. The median overall survival (OS) documented was 46 months (95% CI: 29–101): in particular, 58 months (95% CI: 38-not reached) for NSCLC patients and 18 months (95% CI: 11-not reached) for SCLC patients.

## 4. Discussion

New therapeutic frontiers have significantly improved patient outcomes, enabling better overall survival; however, these drugs are not free from side effects and there remains an unmet need for supportive therapies that can reduce the toxicity of standard anti-cancer drugs and act as adjuvants. In this context, STAT3 inhibitors, and silibinin in particular, have demonstrated documented antitumor activity, acting in different phases of carcinogenesis.

Considering the documented efficacy of these adjuvant molecules, it is of paramount importance to analyze their toxicity profile, in order to demonstrate that they may be administered in combination with standard antineoplastic drugs, without causing significant side effects.

These premises gave rise to our interest in evaluating the tolerability of silibinin in combination with standard therapies in a group of patients with lung cancer. Therefore, our study has demonstrated the excellent tolerance profile of silibinin in patients with brain metastases from lung cancer; indeed, no severe toxic effects were observed and only one patient had diarrhea, which was resolved within a few days, and, moreover, the relationship with silibinin has not been proven, due to the simultaneous use of targeted therapy (tirosin-kinase-inhibitors) that causes the same symptom.

Moreover, based on the evidence that silibinin seems to be an effective antitumor adjuvant in brain cancer, inhibiting growth inhibition and inducting apoptosis, we pointed our attention to brain metastatic lung cancer patients.

In our study, silibinin appears to participate in maintaining quality of life for patients with brain metastasis. From a clinical perspective, silibinin seems to limit the worsening of neurological symptoms and to improve the perception of quality of life, despite the natural progression of tumoral disease. Given that none of the analyses reached the conventional threshold for statistical significance (*p* < 0.05), these findings should be regarded as descriptive and exploratory, highlighting potential clinical trends and confirming the overall absence of measurable changes across timepoints, in the context of metastatic disease progression.

Further analysis could confirm our results and future studies on silibinin properties could open up interesting horizons in the field of adjuvant therapies to standard anti-cancer treatments.

It is also important to remember that silibinin plays an important healing and protective role in the nervous system. Indeed, silibinin might be considered to be a neuroprotective agent against cerebral lesions, through activation of the GAS6/Axl-SIRT1 pathways, improvement in mitochondrial function, reduction in oxidative stress and activation of apoptosis [30,31,32]. This could be an additional role for this molecule in the therapeutic approach to patients with brain metastases.

Some limitations within our study must be observed: the low number of patients enrolled, retrospective nature, lack of randomization and lack of a control arm does not allow us to draw definitive conclusions on the role of silibinin in maintaining QoL. Moreover, while consecutive collection of patients mirrors a real-life population, heterogeneity and lack of standardization may hinder the reproducibility of our results. Additionally, the descriptive design and lack of statistically significant findings show that the results should be interpreted as hypothesis-generating and not as confirmatory evidence of efficacy.

We must also acknowledge that self-administration of questionnaires and lack of blinking represent potential biases in our research.

## 5. Conclusions

To our knowledge, our study is the first collection of a large number of lung cancer patients with brain metastasis taking silibinin as a supplement in association with standard treatments.

Our study shows that combining silibinin with standard lung cancer drugs is very well tolerated, regardless of the type of therapy (biological, immune or chemotherapy).

Furthermore, silibinin allowed our patients to maintain a good quality of life, despite the presence of brain metastases and regardless of the physiological decline in the central nervous system caused by the localization of the disease at this level.

Therefore, silibinin appears to play a role in the management of brain metastases. Further studies will confirm our research and demonstrate the role of this molecule in brain metastases resulting from other neoplasms.

## Figures and Tables

**Table 1:** Characteristics of patients with stage IV lung cancer tumors.

	All Patients	NSCLC	Others
Total gender	86 (100)	73 (100)	13 (100)
Female	45 (52.3)	38 (52.1)	7 (53.8)
Male	41 (47.7)	35 (47.9)	6 (46.2)
Age			
Median [range]	69.5 [40–88]	67 [40–88]	72 [57–82]
ECOG PS			
0	17 (19.8)	17 (23.3)	0 (0.0)
1	41 (47.7)	35 (47.9)	6 (46.2)
2	28 (32.6)	21 (28.8)	7 (53.8)
Smoking status			
Never	27 (31.4)	25 (34.2)	2 (15.4)
Former	40 (46.5)	34 (46.6)	6 (46.2)
Smoker	19 (22.1)	14 (19.2)	5 (38.5)
Histology			
NSCLC ADK	68 (79.1)	68 (93.2)	0 (0.0)
NSCLC Sq	4 (4.7)	4 (5.5)	0 (0.0)
SCLC	10 (11.6)	0 (0.0)	10 (76.9)
LCNEC	3 (3.5)	0 (0.0)	3 (23.1)
NOS Carcinoma	1 (1.2)	1 (1.4)	0 (0.0)
Therapy			
Chemotherapy	20 (23.3)	13 (17.8)	7 (53.8)
Immunotherapy	8 (9.3)	8 (11.0)	0 (0.0)
Chemo-immunotherapy	26 (30.2)	20 (27.4)	6 (46.2)
Biologic	26 (30.2)	26 (35.6)	0 (0.0)
No therapy	6 (7.0)	6 (8.2)	0 (0.0)

**Table 2:** QoL on Sillbrain: differences between baseline and reassessment.

	Baseline	Third Revaluation	*p*-Value *
QLQ-BN20			
Median [range]	19.16 [1.67–66.67]	16.67 [0.00–85.00]	0.43
QLQ-C30: QoL Activity			
Median [range]	78.12 [29.17–100.0]	77.08 [20.00–100.0]	0.82
QLQ-C30: QoL Symptoms			
Median [range]	19.44 [0.00–66.67]	13.89 [0.00–75.00]	0.57
QLQ-C30: Global QoL			
Median [range]	50.00 [0.00–91.67]	66.67 [16.67–100.0]	0.33
EQ5D			
Median [range]	2.00 [0.00–7.00]	2.00 [0.00–9.00]	0.18
QLQ Visual Scale			
Median [range]	52.50 [20.00–90.00]	60.00 [20.00–90.00]	0.6

* *p*-value based on the Wilcoxon test for continuous variables.

## Data Availability

The data were collected anonymously and stored in databases in our departments. However, under Italian law, it is not possible to share our data, as they are unavailable due to privacy or ethical restrictions.
